# An assessment of algorithms to estimate respiratory rate from the electrocardiogram and photoplethysmogram

**DOI:** 10.1088/0967-3334/37/4/610

**Published:** 2016-03-30

**Authors:** Peter H Charlton, Timothy Bonnici, Lionel Tarassenko, David A Clifton, Richard Beale, Peter J Watkinson

**Affiliations:** 1 School of Medicine, King’s College London, UK; 2 Department of Engineering Science, Institute of Biomedical Engineering, University of Oxford, UK; 3 Kadoorie Centre for Critical Care Research and Education, John Radcliffe Hospital, UK; 4 Nuffield Department of Medicine, University of Oxford, UK; peter.charlton@gstt.nhs.uk

**Keywords:** respiratory rate, biomedical signal processing, electrocardiography, photoplethysmography

## Abstract

Over 100 algorithms have been proposed to estimate respiratory rate (RR) from the electrocardiogram (ECG) and photoplethysmogram (PPG). As they have never been compared systematically it is unclear which algorithm performs the best.

Our primary aim was to determine how closely algorithms agreed with a gold standard RR measure when operating under ideal conditions. Secondary aims were: (i) to compare algorithm performance with IP, the clinical standard for continuous respiratory rate measurement in spontaneously breathing patients; (ii) to compare algorithm performance when using ECG and PPG; and (iii) to provide a toolbox of algorithms and data to allow future researchers to conduct reproducible comparisons of algorithms.

Algorithms were divided into three stages: extraction of respiratory signals, estimation of RR, and fusion of estimates. Several interchangeable techniques were implemented for each stage. Algorithms were assembled using all possible combinations of techniques, many of which were novel. After verification on simulated data, algorithms were tested on data from healthy participants. RRs derived from ECG, PPG and IP were compared to reference RRs obtained using a nasal-oral pressure sensor using the limits of agreement (LOA) technique.

314 algorithms were assessed. Of these, 270 could operate on either ECG or PPG, and 44 on only ECG. The best algorithm had 95% LOAs of  −4.7 to 4.7 bpm and a bias of 0.0 bpm when using the ECG, and  −5.1 to 7.2 bpm and 1.0 bpm when using PPG. IP had 95% LOAs of  −5.6 to 5.2 bpm and a bias of  −0.2 bpm. Four algorithms operating on ECG performed better than IP. All high-performing algorithms consisted of novel combinations of time domain RR estimation and modulation fusion techniques. Algorithms performed better when using ECG than PPG. The toolbox of algorithms and data used in this study are publicly available.

## 1. Introduction

Respiratory rate (RR) is a highly informative indicator of physiological state (Braun 1990) and the most sensitive vital sign marker of clinical deterioration (Schein *et al* 1990, Goldhill *et al* 1999, Ridley 2005, Cretikos *et al* 2008). Continuous monitoring of RR provides valuable information regarding patient health status in hospital (Royal College of Physicians 2012), home and community settings (Bonato 2010).

Only a small number of wearable sensors are capable of continuously measuring respiratory rate compared with other key physiological parameters, such as heart rate, temperature and blood oxygen saturation (Hao and Foster 2008). Many utilise impedance plethysmography (Yilmaz *et al* 2010) and inductance plethysmography sensors (Jeong *et al* 2009, Garbino *et al* 2014). These require placement of a tight-fitting band around the wearer’s thorax. Patients find these uncomfortable when worn over prolonged periods (Orphanidou *et al* 2009b). Impedance pneumography (IP), the most commonly used RR sensor in hospitals, is not commonly incorporated into wearable sensors. RR measurement using tri-axial accelerometers remains largely confined to experimental devices (Lapi *et al* 2014).

**Figure 1.**
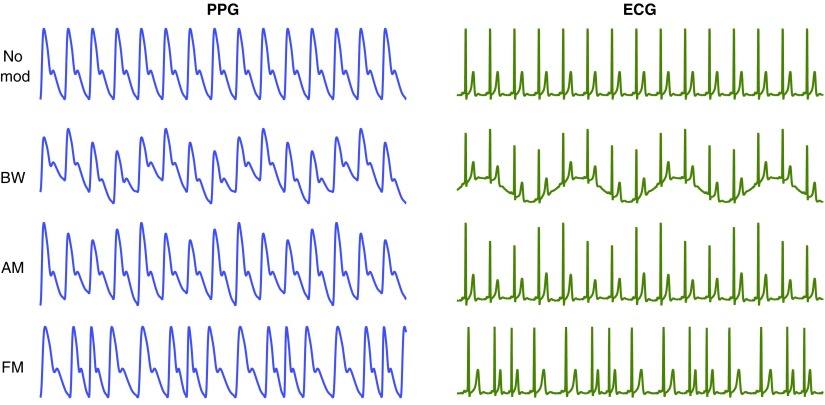
Idealised respiratory modulations of the PPG (left) and ECG (right). From top: no modulation, baseline wander (BW), amplitude modulation (AM), and frequency modulation (FM). Adapted from Addison *et al* (2012) and Pimentel *et al* (2015).

A wide range of existing wearable sensors, including smart watches, record the electrocardiogram (ECG) or pulse oximetry (the photoplethysmogram, PPG) signals. Physiological mechanisms cause both signals to be modulated by respiration. Three types of modulation can be observed, as shown in figure 1: baseline wander (BW), amplitude modulation (AM), and frequency modulation (FM) (Bailon *et al* 2006, Meredith *et al* 2012). Consequently, RR can be estimated from these signals without encumbering the subject with extra sensors. The same techniques can be used to retrospectively derive respiratory rate from ECG or PPG recordings, increasing the value of existing waveform datasets.

Algorithms for estimation of RR from the ECG and PPG were first reported in 1985 and 1992 respectively (Moody *et al* 1985, Lindberg *et al* 1992). Since then over 100 RR algorithms have been proposed to estimate RR from the ECG or PPG. No study has directly compared their performances. Consequently it is unclear to monitor manufacturers and end users which, if any, algorithms perform sufficiently well for their application.

A number of limitations prevent meta-analysis of existing studies to derive an overall ranking of performance. Firstly, several statistical issues impede comparison. Different papers report different statistical measures of measurement accuracy. Few papers take into account the repeated measures structure of the data from validation studies of continuous monitors. The number of participants in many studies is small. Secondly, when proposing novel algorithms, authors have compared the novel algorithm against their own implementation of an existing algorithm. Differences between authors’ implementations of a particular algorithm might lead to significant differences in performance. Thirdly, some studies have assessed algorithms on data acquired from mechanically ventilated subjects. Mechanical ventilation, such as occurs in anaesthetised patients, is known to affect the physiology of both ECG and PPG modulations (Hirsch and Bishop 1981, Johansson and Strömberg 2000). Results from these studies are not necessarily representative of performance in spontaneously breathing subjects.

The primary aim of this study was to determine how closely algorithms agreed with a gold standard RR measure when operating under ideal conditions. Secondary aims were: (i) to compare algorithm performance with IP, the clinical standard for continuous respiratory rate measurement in spontaneously breathing patients; (ii) to compare algorithm performance when using ECG and PPG; and (iii) to provide a toolbox of algorithms and data to allow future researchers to conduct reproducible comparisons of algorithms (available at: http://peterhcharlton.github.io/RRest).

## 2. Respiratory rate algorithms

**Figure 2.**
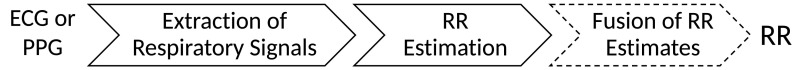
The three stages of a respiratory rate (RR) algorithm, which estimates RR from either the ECG or the PPG. The dashed stage is optional. Adapted from Pimentel *et al* (2015).

RR algorithms can be divided into three stages, as illustrated in figure 2. Two of the stages, namely ‘extraction of respiratory signals’ and ‘RR estimation’, are compulsory whereas ‘fusion of RR estimates’ is optional. An RR algorithm can be constructed by selecting a technique for each stage. Algorithms can use either the ECG or PPG as an input signal, except those which use measures specific to the QRS complex or PPG pulse.

We implemented several techniques for each stage. These are summarised in tables 1–3. We did not include techniques which rely upon simultaneous recording of ECG from multiple leads as many wearable sensors do not provide multi-lead ECG recordings. All possible RR algorithms which could be constructed using these techniques were assessed. The implementations of each stage are now described.

**Table 1.** Techniques for extraction of respiratory signals.

Abbr.	Technique
	*Filter-Based,* ${{X}_{\text{A}1,\ldots,4}}$
*X*_A1_	BW: Band-pass filter between 4 and 60 bpm (Lindberg *et al* 1992).
*X*_A2_	AM: The maximum amplitude of the Continuous Wavelet Transform (CWT) within plausible cardiac frequencies (30–220 beats per minute) (Addison and Watson 2004).
*X*_A3_	FM: The frequency corresponding to the maximum amplitude of the CWT within plausible cardiac frequencies (Addison and Watson 2004).
*X*_A4_	BW, AM, FM: Filter using the centred-correntropy function (CCF) (Garde *et al* 2014).
	*Feature-Based,* ${{X}_{\text{B}1,\ldots,8}}$
*X*_B1_	BW: mean amplitude of troughs and proceeding peaks.
*X*_B2_	AM: difference between the amplitudes of troughs and proceeding peaks (Karlen *et al* 2013).
*X*_B3_	FM: time interval between consecutive peaks (Orphanidou *et al* 2013, Karlen *et al* 2013).
*X*_B4_	BW: mean signal value between consecutive troughs (Ruangsuwana *et al* 2010).
*X*_B5_	BW, AM: peak amplitude (Karlen *et al* 2013).
*X*_B6_	BW, AM: trough amplitude (Ruangsuwana *et al* 2010).
*X*_B7_	FM: QRS duration (Rajkumar and Ramya 2013). Q and S waves were identified as the minima immediately before and after the R wave (Ruangsuwana *et al* 2010).
*X*_B8_	AM, FM: QRS area (Sobron *et al* 2010), defined as the integral of the ECG between Q and S waves after subtraction of a baseline linearly interpolated between Q and S waves.
*X*_B9_	BW: Kernel principal component analysis using a radial basis function, with the variance of the Gaussian kernel determined by maximising the difference between the first eigenvalue and sum of the remainder (Widjaja *et al* 2012).
*X*_B10_	FM: PPG pulse width estimated using a wave boundary detection algorithm (Lázaro Plaza 2015).

**Table 2.** Techniques for respiratory rate estimation.

Abbr.	Technique
	*Frequency-based,* ${{E}_{\text{F}1,\ldots,5}}$
*E*_F1_	Fast Fourier transform spectral analysis (Karlen *et al* 2013).
*E*_F2_	Auto-regressive spectral analysis (Thayer *et al* 2002) using model order 8 (Orphanidou *et al* 2013).
*E*_F3_	Auto-regressive spectral analysis using the median spectrum for model orders 2–20 (Shah *et al* 2015).
*E*_F4_	Auto-regressive all-pole modelling (order 8), with the highest magnitude pole selected as the respiratory pole (Fleming *et al* 2008).
*E*_F5_	Auto-regressive all-pole modelling (order 8), with the lowest frequency pole selected as the respiratory pole (Fleming and Tarassenko 2006).
*E*_F6_	Find periodicity using the autocorrelation function (Schäfer and Kratky 2008).
*E*_F7_	Spectral analysis using the Welch periodogram (Lázaro Plaza 2015).
	*Time-domain breath detection,* ${{E}_{\text{T}1,\ldots,5}}$
*E*_T1_	Breath detection by peak detection (Shah 2012).
*E*_T2_	Breath detection by positive gradient zero-crossing detection (Johansson 2003).
*E*_T3_	Breath detection by combined peak and trough detection (Fleming 2010): elimination of peaks less than the mean, and troughs greater than the mean; elimination of peaks (and troughs) within 0.5s of the previous peak (or trough); elimination of peaks (and troughs) which are immediately followed by a peak (or trough).
*E*_T4_	Breath detection using ‘Count-orig’ (Schäfer and Kratky 2008): detrend; detect peaks and troughs; define a threshold as 0.2 times the 75th percentile of peak values; ignore peaks with an amplitude below this threshold; identify valid breaths as consecutive peaks separated by only one trough with an amplitude less than zero.
*E*_T5_	Breath detection using ‘Count-adv’ (Schäfer and Kratky 2008): detrend; detect peaks and troughs; define a threshold as 0.3 times the 75th percentile of amplitude differences between consecutive extrema; eliminate the pair of extrema with the smallest amplitude difference if this is below the threshold; repeat until no more pairs can be eliminated; remaining peaks represent breaths.

**Table 3.** Techniques for fusion of RR estimates.

Abbr.	Technique
	*Modulation,* ${{F}_{\text{M}1,\ldots,4}}$
*F*_M1_	Smart fusion (Karlen *et al* 2013): RRs estimated from BW, AM and FM respiratory signals (${{X}_{\text{B}1,2,3}}$) are quality assessed. If their standard deviation is $\leqslant 4$ bpm then RR is estimated as the mean, otherwise no RR is output.
*F*_M2_	Spectral peak-conditioned averaging (Lázaro Plaza 2015): Frequency spectra calculated from BW, AM and FM respiratory signals (${{X}_{\text{B}1,2,3}}$) using the Welch periodogram (*F*_*T*7_) are fused to give a mean spectrum. Only those spectra for which a certain proportion of spectral power is contained within a frequency range centred on the frequency corresponding to the maximum spectral power are included (a modification of the reported method). RR is estimated as the frequency corresponding to the maximum power in the mean spectrum.
*F*_M3_	Pole magnitude criterion (Orphanidou *et al* 2013): The respiratory pole is chosen as the highest magnitude pole obtained from auto-regressive spectral analysis of BW, AM and FM respiratory signals (${{X}_{\text{B}1,2,3}}$).
*F*_M4_	Pole ranking criterion (Orphanidou *et al* 2009a): The pair of highest magnitude poles obtained from auto-regressive spectral analysis of BW, AM and FM respiratory signals (${{X}_{\text{B}1,2,3}}$) with the greatest pole ranking criterion (PRC) is selected. $\text{PRC}=\overline{{{m}_{i,j}}}/d{{\theta}_{i,j}}^{2}$, where $\overline{{{m}_{i,j}}}=\left({{m}_{i}}+{{m}_{j}}\right)/2$ and $d{{\theta}_{i,j}}=\,|{{\theta}_{i}}-{{\theta}_{j}}|$, for $i,j=1,\ldots,N$, where N is the number of poles calculated. *θ* and *m* are the pole angles and magnitudes respectively. RR is estimated from the mean frequency corresponding to the selected pair.
	*Temporal, F*_*T1*_
*F*_T1_	Temporal smoothing (Lázaro *et al* 2013): estimated RRs, $\text{R}{{\text{R}}_{\text{est}}}$, are smoothed to give the final RR, $\text{R}{{\text{R}}_{i}}$, using $\text{R}{{\text{R}}_{i}}=0.2\text{R}{{\text{R}}_{\text{est}}}+0.8\text{R}{{\text{R}}_{i-1}}$.

### 2.1. Extraction of respiratory signals

The extraction stage derives a respiratory signal (a time series dominated by respiratory modulation) from the original signal (ECG or PPG). The initial step, common to all techniques, was elimination of very low frequencies. This was achieved using a high-pass filter with  −3 dB cutoff frequency of 4 breaths per minute (bpm). Intermediate steps were determined by whether a filter- or feature-based extraction technique was being used, as described below. The final step, again common to all techniques, was elimination of non-respiratory frequencies from the extracted signal using a band-pass filter (with  −3 dB cutoff frequencies of 4 and 60 bpm).

Filter-based extraction consisted of filtering the signal using one of the four techniques in table 1, ${{X}_{\text{A}1,\ldots,4}}$. Feature-based extraction consisted of extracting a time series of beat-by-beat feature measurements through several steps as follows. Very high frequencies were eliminated using low-pass filters with  −3 dB cutoffs of 100 and 35 Hz for the ECG and PPG respectively. An additional 50 Hz notch filter was used to eliminate mains interference in the ECG. Beat detection was performed on the ECG using a QRS detector based upon the algorithm of Pan and Tompkins (1985) and Hamilton and Tompkins (1986), and on the PPG using the incremental-merge segmentation (IMS) algorithm (Karlen *et al* 2012). R-waves and pulse peaks were detected as the maxima at or between detected beats. QRS troughs were detected as the minima within the 0.10 s prior to R-waves (Ruangsuwana *et al* 2010), and pulse troughs as the minima between pulse peaks (Johansson 2003). One of the beat-by-beat features, ${{X}_{\text{B}1,\ldots,10}}$, was obtained as described in table 1. Features derived from ectopic beats were eliminated using the algorithm described in Mateo and Laguna (2003). The irregularly sampled signal of features was resampled at 5 Hz using linear interpolation (Karlen *et al* 2013). Finally, very low frequencies were eliminated using a high-pass filter with  −3 dB cutoff of 4 bpm.

### 2.2. Respiratory rate estimation

Estimation of RR from a respiratory signal was performed using each of the techniques ${{E}_{\text{F}1,\ldots,7}}$ and ${{E}_{\text{T}1,\ldots,5}}$ in table 2. When using spectral analysis (${{E}_{\text{F}1,2,3,7}}$) the RR was identified as corresponding to the frequency of the spectral peak with greatest magnitude between 4 and 60 bpm. When using breath detection methods (${{E}_{\text{T}1,\ldots,5}}$) the estimated RR was calculated as the mean breath duration.

### 2.3. Fusion of RR estimates

The fusion stage has received particular attention recently due to the improvements in algorithm performance observed with its use (Karlen *et al* 2013). Four modulation fusion methods, ${{F}_{\text{M}1,\ldots,4}}$ in table 3, were optionally used to fuse simultaneous RR estimates corresponding to each modulation, extracted using ${{X}_{\text{B}1,2,3}}$. Temporal fusion, *F*_T1_, was optionally used to smooth successive RR estimates obtained from the same individual. It may be used with or without preceding modulation fusion.

## 3. Methods

### 3.1. Verification of technique implementations

We verified that techniques had been implemented successfully by testing on simulated data. Techniques were combined into algorithms as described above. All algorithms were assessed on both simulated ECG and PPG, except where the respiratory extraction technique was signal-specific: *X*_*B*7_ and *X*_*B*8_ only operate on ECG, and *X*_*B*10_ only operates on PPG.

Simulated data, such as those shown in figure 1, were generated as follows. Single ECG and PPG beats were replicated to generate simulated signals consisting of trains of ECG and PPG beats, each of 210 s duration sampled at 500 Hz. Each of these two simulated signals was modulated separately to mimic each respiratory modulation (BW, AM, and FM), leading to six modulated signals. The modulations were repeated for a range of physiologically plausible heart rates (HRs) and RRs. A first set of 35 simulated signals was generated for each modulation by holding RR constant at 18 bpm whilst the HR was varied from 30 to 200 bpm at intervals of 5 bpm. A second set of 29 simulated signals was simulated by holding the HR constant at 80 bpm whilst RR was varied from 4 to 60 bpm at intervals of 2 bpm. Hence, a total of 192 simulated ECG and PPG signals were generated with which to verify algorithm implementations.

A technique was considered to be successfully implemented if over half of the algorithms containing that technique were acceptably accurate. ‘Acceptably accurate’ was defined as an absolute error of  ⩽1 bpm for at least 50% of the simulated signals for any of the three modulations, on either the ECG or PPG. The threshold of 50% was chosen to account for algorithms which perform well for subsets of the trialled HR and RR combinations, but fail under extreme physiological conditions. The correlation between performance on the simulated data and real data was unknown at the time of verification. However, it seemed reasonable to assume that algorithms which performed extremely poorly on simulated data would not perform well on real data.

A total of 370 algorithms were implemented. 314 algorithms (85%) met our acceptability criteria. Algorithms containing either *E*_*F*5_ or *X*_*B*10_ were excluded. *E*_*F*5_ performed poorly at RRs outside of 12–20 bpm, which may be due to its bias towards identifying lower frequencies as the RR. *X*_*B*10_ variably detected the end of the PPG pulse as either the time of the minimum immediately before the diastolic peak, or the time of the diastolic peak, causing inaccuracies.

### 3.2. Participants

Healthy adults aged between 18 and 40 years of age participated as part of the VORTAL study (National Clinical Trial 01472133). Ethical approval was obtained from the London Westminster Research Ethics Committee (11/LO/1667). Exclusion criteria were the presence of co-morbidities or medications that might significantly affect the functioning of the cardiac, respiratory and autonomic nervous systems.

### 3.3. Signal acquisition

Lead II ECG, PPG recorded from a finger probe, and oral-nasal pressure signals were acquired using a 1902 amplifier, a Power 1401 analogue-to-digital converter and Spike2 v.7.09 acquisition software (all Cambridge Electronic Design, Cambridge, UK). PPG was transduced using an MLT1020FC infrared reflection plethysmograph (AD Instruments, CO Springs, New Zealand). Oral-nasal pressure was transduced using an Ultima Dual Airflow differential pressure transducer (Braebon Medical Corporation, Kantata, ON, Canada) connected to a P1300 Pro-Flow oronasal cannula (Philips Respironics, Murrysville, PA, USA). Signals were sampled at 500 Hz.

Thoracic IP RRs were acquired at 1 Hz using an IntelliVue MP30 monitor (Philips Medical Systems, Boeblingen, Germany) and ixTrend acquisition software (v.2.0.0 Express, Ixellence GmbH, Wildau, Germany).

### 3.4. Data recording procedures

We prepared the subject’s skin prior to application of ECG electrodes in the Mason-Likar configuration. We shaved excess chest hair and cleansed the skin with an alcohol wipe before removing dead skin using 10–15 light strokes with abrasive skin preparation tape. The PPG probe was placed on the right hand.

Two 10 min recordings were acquired from each subject laid supine in a room maintained between 22 and 25 °C. After the first recording subjects were asked to run on a treadmill until their HR reached 85% of their age-predicted maximum, as described in Gibbons *et al* (1997). The second recording was acquired immediately following exercise. The post-exercise recordings contained a wider range of HRs and RRs than the pre-exercise recordings.

### 3.5. Respiratory rate estimation from ECG, PPG and IP

**Figure 3.**
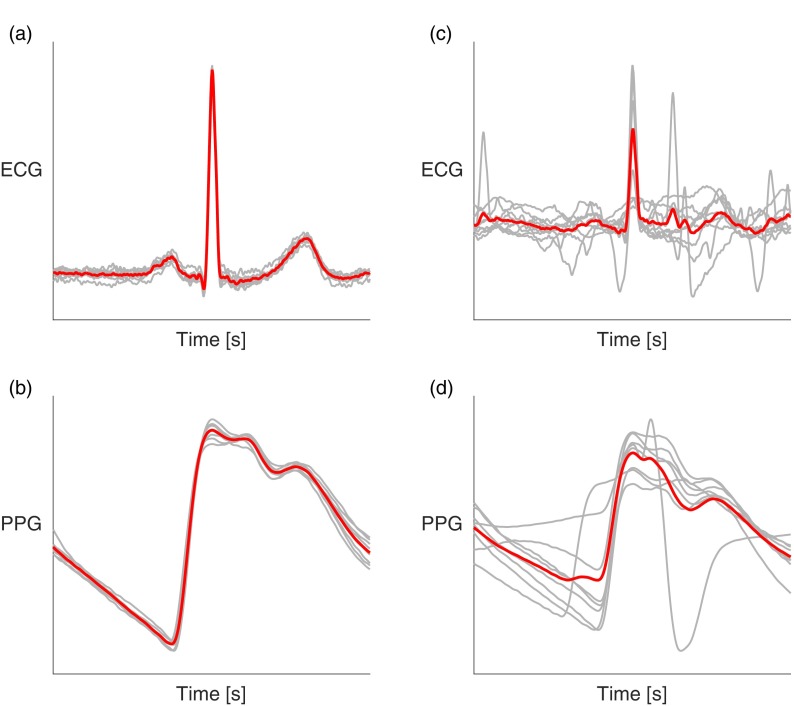
ECG and PPG signal quality assessment: Signals were segmented into windows, and the correlation between individual beats (thin grey lines) in a window and the window’s average beat template (thick red line) was calculated. If the correlation was below an empirically determined threshold then the segment was deemed to be of low quality, as described in Orphanidou *et al* (2015). (a) and (b) show high quality windows, whereas (c) and (d) show low quality windows.

Respiratory signals derived from the ECG or PPG were segmented into adjacent windows of 32 s. Signal quality was assessed using the algorithm described in Orphanidou *et al* (2015). Firstly, the algorithm detects beats in the signal. Secondly, beat-to-beat intervals and HRs are measured. If either are physiologically implausible, then the window is deemed to be of low quality. For each high quality window a template beat is constructed. The correlation between individual beats and the template is assessed. If the correlation is below an empirically determined threshold, then the window is deemed to be of low quality, as shown in figure 3. Windows labelled as low quality were excluded from the analysis.

For each window RR was estimated using all possible combinations of algorithms and input signal. IP RRs were also calculated, based on the readings provided by the monitor. They were estimated as the mean of all IP RR values during that window.

### 3.6. Reference respiratory rate calculation

Reference RRs were calculated from the oral-nasal pressure signal using a custom breath-detection algorithm. The signal was band-pass filtered with  −3 dB cutoff frequencies of 4 and 60 bpm. It was segmented into the same 32s windows as used for the ECG and PPG. The signal was normalised within each window to have a mean of 0 and standard deviation of 1. Inhalations were identified as positive-gradient crossings of a specified threshold, *k*. A threshold of *k*  =  0.42 was determined by minimising the difference between the value of 2SD (explained below) derived from the comparison of the reference RRs to expert breath annotations on the first 10 subjects. The performance of the reference RR calculator was then assessed against a second expert’s breath annotations on the subsequent 10 subjects. It had a bias of 0.0 bpm and 2SD of 1.3 bpm. Windows in which the pressure signal had a low signal-to-noise ratio were excluded from the analysis. The threshold for exclusion was chosen to eliminate windows in which breaths could not be identified visually.

### 3.7. Statistical analysis

Agreement between each algorithm and the reference was assessed using the limits of agreement (LOA) technique. The bias, 95% LOAs, within subject variability (WSV) and between subject variability (BSV) were estimated using a random effects model to account for repeated measures in the data. Following Carstensen *et al* (2008) the random effects model used was

1 \begin{eqnarray*}\begin{array}{*{20}{l}}&amp;y_{mir} = \alpha_m + \mu_i + a_{ir} + c_{mi} + e_{mir} \quad \\ &amp; \quad a_{ir} \sim \mathcal{N}(0,\omega^2) \quad c_{mi} \sim \mathcal{N}(0,\tau^2) \quad e_{mir} \sim \mathcal{N}(0,\sigma_m^2) \quad ,\end{array}\end{eqnarray*}

where *y* is the measurement, *m* is the method, *i* is the subject and *r* is the replicate. ${{\alpha}_{m}}$ is a fixed effect representing a method’s constant underlying bias, ${{\mu}_{i}}$ is the mean bias for a particular subject, *a*_*ir*_ is a random effect accounting for the similarity of measurements within a subject, *c*_*mi*_ is a random effect related to the BSV (${{\tau}^{2}}$), and *e*_*mir*_ is the residual error related to the WSV of the method ($\sigma _{m}^{2}$). The bias was calculated for each algorithm as ${{\alpha}_{\text{reference}}}-{{\alpha}_{\text{algorithm}}}$, and the 95% LOAs as mean bias  ±2 standard deviations (2SD) where

2 \begin{eqnarray*}2\text{SD}=2\sqrt{2{{{\hat{\tau}}}^{2}}+\hat{\sigma}_{\text{ref}}^{2}+\hat{\sigma}_{\text{alg}}^{2}}.\end{eqnarray*}

A second measure of agreement, the coverage probability (CP$_{\delta}$) is also reported. The CP$_{\delta}$ is the probability of measurement error falling within pre-defined bounds, *δ*. The non-parametric form of CP, expressed as a percentage, was calculated using the empirical cumulative distribution of the absolute error (Barnhart *et al* 2007) with *δ* set at 2 bpm.

The effect of the input signal on 2SD and bias was assessed using the Wilcoxon signed-rank test for those algorithms which could operate on both ECG and PPG.

### 3.8. Ranking criteria

Methods were ranked by sorting first by 2SD and then by absolute bias. Values of 2SD and absolute bias were rounded to one decimal place for sorting. 2SD, a statistic related to precision, was prioritised over bias when ranking since one is commonly more concerned with tracking trends in RR than absolute values when using wearable sensors for continuous monitoring. Furthermore, bias can be corrected by calibration if an instrument is precise.

## 4. Results

### 4.1. Recruitment and data characteristics

**Figure 4.**
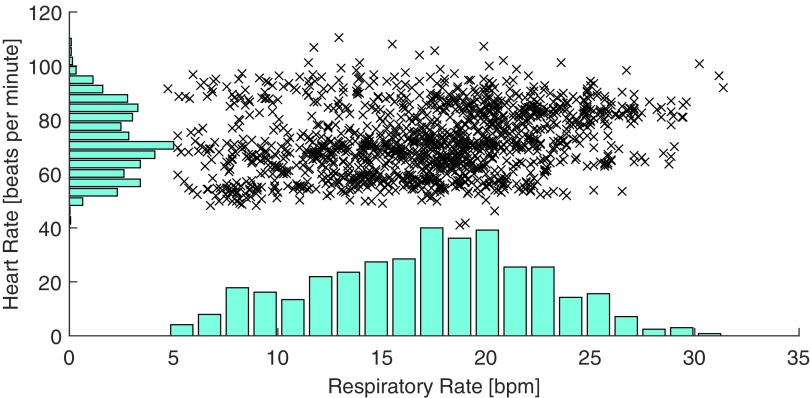
The ranges of respiratory rates (RRs), measured in breaths per minute (bpm), and heart rates (HRs) in the dataset.

Data were acquired from 45 subjects. Five subjects were excluded since their recordings were incomplete, and one due to the development of an arrhythmia following exercise. 39 subjects’ data were analysed. The median (lower, upper quartiles) age of analysed subjects was 29 (26, 32) years. Their median BMI was 23 (21, 26) kgm^−2^. 21 (54%) were female. Data from each subject contained a median of 40 (37, 41) windows. The oral-nasal pressure signal was of high quality in 40 (37, 40) windows. Of these, ECG and PPG were of high quality in 37 (34, 40) and 36 (34, 39) windows respectively. The ranges of RR and HR in the dataset were 5–32 bpm and 41–111 beats per minute respectively. The distributions of RR and HR are shown in figure 4.

### 4.2. Algorithm performance

Of the 314 algorithms assessed, 270 could operate on both ECG and PPG and 44 were specific to the ECG. Therefore 584 algorithm-signal combinations were tested. The random effects model did not converge for 34 of these, all of which used *E*_F4_. Inspection revealed minimal correlation between the reference and estimated RRs in these instances. These combinations were excluded from further analysis.

**Figure 5.**
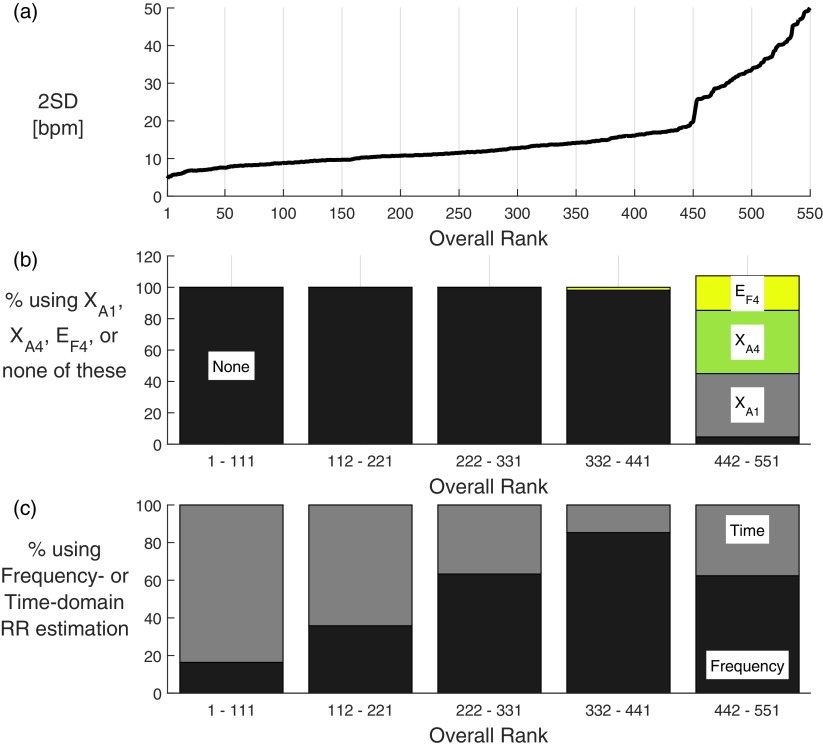
(a) shows the precision (2SD) of each possible combination of algorithm and input signal, with lower values indicating better performance; (b) shows the percentage of combinations which used *X*_A1_, *X*_A4_, *E*_F3_, or none of these techniques (bars may exceed 100% since an algorithm can use more than one of the techniques); (c) shows the percentage of combinations which used frequency- or time-domain RR estimation techniques.

The values of 2SD of the remaining algorithm-signal combinations ranged from 4.7 bpm at best to 50.1 bpm at worst, as shown in figure 5(a). The top ranked method was ${{X}_{\text{B}1,2,3}}{{E}_{\text{T}4}}{{F}_{\text{M}1}}\left(\text{ECG}\right)$, whose measurements had a bias of 0.0 bpm, 95% LOA of  −4.7 to 4.7 bpm and CP_2_ of 80.5%. IP was ranked 5th with a bias of  −0.2 bpm, 95% LOA of  −5.6 to 5.2 bpm and CP_2_ of 76.0%. Performance metrics for the top 10 algorithms using ECG and PPG are shown in table 4. Metrics for all algorithm-signal combinations are reported in the supplementary material (stacks.iop.org/PM/37/610/mmedia).

**Table 4.** Performances of the ten highest ranked algorithms for the ECG and PPG, and of IP-Derived RR: Ranked by 2SD followed by absolute bias.

Signal	Algorithm	Overall rank	2SD (bpm)	Bias (bpm)	95% LOA (bpm)	Proportion of windows with RR estimate (%)	CP_2_ (%)
IP	Clinical monitor	5	5.4	−0.2	−5.6 to 5.2	100.0	76.0

	${{X}_{\text{B}1,2,3}}$*E*_T4_*F*_M1_	1	4.7	0.0	−4.7 to 4.7	73.8	80.5
	${{X}_{\text{B}1,2,3}}$ ${{E}_{\text{T}2}}$ *F*_M1_	2	5.2	1.4	−3.8 to 6.4	72.3	72.6
	${{X}_{\text{B}1,2,3}}$${{E}_{\text{T}5}}$ ${{F}_{\text{M}1}}$	3	5.2	2.0	−3.3 to 7.2	75.4	69.1
ECG	${{X}_{\text{B}1,2,3}}$${{E}_{\text{T}3}}$ ${{F}_{\text{M}1}}$	4	5.3	1.4	−3.8 to 6.7	72.5	73.0
	*X*_B2_${{E}_{\text{T}2}}$	6	5.6	−0.2	−5.8 to 5.4	100.0	75.2
	*X*_B2_${{E}_{\text{T}3}}$	7	5.7	−0.2	−5.9 to 5.4	100.0	74.3
	*X*_B2_${{E}_{\text{T}2}}$ ${{F}_{\text{T}1}}$	8	5.7	−0.2	−6 to 5.5	100.0	69.3
	*X*_B2_${{E}_{\text{T}5}}$	9	5.7	0.5	−5.2 to 6.3	100.0	74.9
	*X*_B2_${{E}_{\text{T}3}}$ ${{F}_{\text{T}1}}$	10	5.8	−0.2	−6.0 to 5.6	100.0	69.8
	${{X}_{\text{B}1,2,3}}$${{E}_{\text{T}4}}$${{F}_{\text{M}1}}$${{F}_{\text{T}1}}$	11	5.9	0.0	−5.9 to 6.0	100.0	66.6

	${{X}_{\text{B}1,2,3}}$${{E}_{\text{T}4}}$ ${{F}_{\text{M}1}}$	15	6.2	1.0	−5.1 to 7.2	54.2	71.5
	${{X}_{\text{B}1,2,3}}$${{E}_{\text{T}1}}$ ${{F}_{\text{M}1}}$	17	6.5	−1.0	−7.5 to 5.5	62.1	62.1
	${{X}_{\text{B}1,2,3}}$${{E}_{\text{T}1}}$ *F*_M1_${{F}_{\text{T}1}}$	35	7.0	−1.3	−8.3 to 5.7	100.0	54.2
PPG	*X*_B2_${{E}_{\text{T}5}}$ ${{F}_{\text{T}1}}$	46	7.5	3.0	−4.5 to 10.5	100.0	44.3
	*X*_B5_${{E}_{\text{T}1}}$ ${{F}_{\text{T}1}}$	48	7.6	0.7	−6.9 to 8.3	100.0	57.0
	*X*_B2_${{E}_{\text{T}2}}$ ${{F}_{\text{T}1}}$	53	7.6	2.7	−4.9 to 10.3	100.0	47.2
	${{X}_{\text{B}1,2,3}}$ ${{E}_{\text{T}4}}$ ${{F}_{\text{M}1}}$ ${{F}_{\text{T}1}}$	54	7.8	1.1	−6.8 to 8.9	97.3	57.2
	${{X}_{\text{B}1,2,3}}$ ${{E}_{\text{T}5}}$ ${{F}_{\text{M}1}}$	55	7.8	3.8	−4.0 to 11.5	70.9	49.8
	*X*_B2_${{E}_{\text{T}4}}$ ${{F}_{\text{T}1}}$	56	7.9	0.3	−7.7 to 8.2	100.0	60.5
	${{X}_{\text{B}1,2,3}}$ ${{E}_{\text{T}2}}$ ${{F}_{\text{M}1}}$ ${{F}_{\text{T}1}}$	58	7.9	3.7	−4.2 to 11.5	100.0	60.5

*Note*: Definitions of the techniques are provided in tables 1–3.

Four algorithm-signal combinations were more precise than IP. These combinations all used a time-domain RR estimation technique and a modulation fusion technique, and operated on the ECG. They provided RR estimates for between 72.3 and 75.4% of windows. In contrast, IP and the next best-performing combinations provided estimates for all windows.

There was a sharp increase in values of 2SD for algorithm-signal combinations ranked in the lowest quintile, as shown in figure 5(a). Algorithms within this quintile contained the *X*_A1_, *X*_A4_ or *E*_F4_ components suggesting that these components were responsible for poorer performance (figure 5(b)). Figure 5(c) shows that more of the better-performing combinations used a time-domain technique, and more of the worse-performing combinations used a frequency-domain technique.

### 4.3. Comparison between ECG and PPG

Of the 283 algorithms which could operate on both ECG and PPG, 30 were excluded because the random effects model did not converge when operating on at least one of the signals. Of the remaining 253 algorithms, 161 (63.6%) were more precise when using ECG and 92 (36.4%) were more precise when using PPG. There was a statistically significant median decrease in the 2SD of 0.8 bpm when algorithms operated on ECG (11.6 bpm) compared to when they operated on PPG (12.4 bpm), *z*  =  −4.024, *p*  =  0.001.

${{X}_{\text{B}1,2,3}}$ ${{E}_{\text{T}4}}$ *F*_M1_ gave the most precise estimates for both ECG and PPG-based methods. When operating on PPG it ranked 15th overall. The algorithms which were ranked in the top 10 when using ECG and those in the top 10 when using PPG shared common features. All used feature-based extraction techniques (${{X}_{\text{B}1,\ldots,5}}$) and time-domain breath-detection estimation techniques (${{E}_{\text{T}1,\ldots,5}}$). All except three used either smart fusion (*F*_M1_, a type of modulation fusion) or temporal fusion (*F*_T1_).

## 5. Discussion

To the best of our knowledge, this study represents the most comprehensive assessment of algorithms for estimation of RR from the ECG and PPG to date. A total of 314 algorithms, initially verified on simulated data, were assessed. In contrast, previous studies have assessed the performances of between 4 and 12 algorithms (Fleming and Tarassenko 2006, Schäfer and Kratky 2008, Nemati *et al* 2010, Ruangsuwana *et al* 2010, Lázaro *et al* 2013, Garde *et al* 2014).

We assembled all possible combinations of the 31 implemented techniques (tables 1–3) rather than restricting analysis to previously published algorithms. This approach produced high performance algorithms consisting of novel combinations of techniques. The best-performing algorithms combined smart fusion (*F*_M1_) with a time-domain breath-detection RR estimation technique (${{E}_{\text{T}1,\ldots,5}}$). This approach accounted for the majority of the top ranked algorithms. To our knowledge this combination of techniques has not been reported before. A further novelty was the demonstration that smart fusion worked well with ECG. Previously it had only been applied to PPG (Karlen *et al* 2013).

Four algorithms, operating on the ECG, were found to be more precise than IP. IP is the clinical standard for continuous monitoring of RR in a hospital ward environment. Our results suggest that ECG-based algorithms could be sufficiently precise for clinical decision making. However, further work is required to determine whether algorithms perform sufficiently well for use in specific clinical settings.

Algorithms performed better when using the ECG than when using the PPG. This may be caused by differences in the physiological mechanisms resulting in the respiratory modulations in each signal. AM and BW in the ECG are caused by changes in the orientation of the electrical axis of the heart relative to the electrodes, and changing thoracic impedance (Bailon *et al* 2006). In the PPG these modulations result from changes in venous return, stroke volume, and arterial blood pressure (Meredith *et al* 2012). In our young healthy population the signal to noise ratio produced by the modulations may be higher in the ECG than the PPG. However, this might not be the case in other populations, particularly where thoracic wall movement is restricted.

The vast majority of the top-ranked algorithms used a fusion component. Some used smart fusion (*F*_M1_), which combines measures of all three respiratory modulations. Smart fusion increased precision at the expense of decreasing the frequency of RR estimates and increasing computational requirements. Others used temporal fusion (*F*_T1_), which maintained a high frequency of estimates at the expense of precision.

All of the top-ranked algorithms used time-domain breath-detection techniques to estimate RR (${{E}_{\text{T}1,\ldots,5}}$). Time-domain techniques do not require a respiratory signal to be quasi-stationary, unlike frequency-domain techniques. This may explain their superior performance. Algorithms incorporating one particular frequency-domain technique, *E*_F4_, performed poorly in this study. This technique used auto-regressive all-pole modelling with a fixed model order of 8, as described in Orphanidou *et al* (2013). Model order selection is known to affect the performance of auto-regressive models. Consequently, automated methods for choosing the model order for RR estimation have been described previously, including the minimum description length method (Garde *et al* 2014), the Akiake criteria (Nemati *et al* 2010), or the optimal parameter search criterion (Lee and Chon 2010). Further developments of frequency-domain RR estimation techniques such as automated model order selection may improve their performance.

### 5.1. Limitations

We cannot claim that all our implementations match those described previously. In some cases this is because algorithms have not been described in sufficient detail to be replicated exactly (Addison *et al* 2015). In other cases we have omitted algorithm-specific tuning steps which did not readily fit into our framework. However, the implementations of each technique were verified using simulated data, ensuring that poor performance was not due to incorrect implementation. By providing the source code of our algorithms we have provided the opportunity for others, including the authors of the original implementations, to verify our work and suggest improvements.

Although we have attempted to be comprehensive, we cannot claim to have implemented all techniques reported in the literature. This was not feasible due to the number of techniques previously proposed. We welcome contributions of new techniques for inclusion in future versions of the toolkit.

Performance rankings are approximate. In common with most statistical measures of agreement, calculation of the LOA depends on the distribution of measurement differences being normal and constant across the range of RRs. The distribution of RR measurement differences varies between algorithms, with some algorithms violating these assumptions to a greater degree than others. Therefore the LOA may be more accurately estimated for some than others. However, the estimation error is unlikely to be large enough to change the conclusions of our study.

While the 2SD provides a measure of the trustworthiness of a single measurement in isolation, the CP_2_ provides a descriptor of the algorithms’ typical performance. Algorithms with a high CP_2_ but relatively large value of 2SD may merit further investigation. Such algorithms performed very well for a large proportion of the data and extremely poorly for the remainder. Modified versions of these algorithms might perform very well if it is possible to mitigate against the factors causing occasional poor performance.

The results cannot be directly extrapolated to other monitoring scenarios, such as during exercise, nor other populations, such as elderly or unwell subjects. We designed the study to demonstrate the best possible results which can be obtained from RR algorithms using ECG and PPG signals. To do so we recorded data from young healthy volunteers at rest using high-fidelity recording equipment and pre-processed signals to exclude artifact. In a clinical setting, for instance, the patient population and signal acquisition equipment are different. These differences may significantly affect algorithm performance.

### 5.2. Future work

Future studies should examine the effects of the subject population, artifact, and recording equipment on algorithm performance. This will determine the generalisability of the conclusions of this study. It will also be valuable to assess the utility of RR algorithms in a wider range of monitoring scenarios, including in the clinical setting.

This study indicates promising directions for future algorithm development. The smart fusion technique (*F*_M1_) improved algorithm performance in this study. This technique weights RR estimates derived from each respiratory modulation equally. However, age and comorbidities may influence the strength of modulations. For instance, respiratory sinus arrhythmia (which causes FM) and chest wall excursion (which is linked to BW and AM) both diminish with age (Moll and Wright 1972, O’Brien *et al* 1986). Weighting the fusion according to the strength of each modulation’s manifestation in a particular subject’s data may improve performance.

In this study we have not investigated the computational requirements of algorithms. This may be important when deciding whether algorithms are suitable for use in real-time, power constrained settings, such as in wearable sensors in the wellbeing sector. In these settings the choice of algorithm may be strongly determined by computational capacity as well as precision.

## 6. Conclusion

We assessed the performances of 314 algorithms for estimation of RR from the ECG and PPG under ideal operating conditions including many novel algorithms. The top ranked method was ${{X}_{\text{B}1,2,3}}\,{{E}_{\text{T}4}}\,{{F}_{\text{M}1}}\left(\text{ECG}\right)$. Four algorithms performed better than IP, the current clinical standard for non-invasive RR measurement, when operating on the ECG. This suggests that ECG-based algorithms may perform sufficiently well for use in particular clinical scenarios.

The conclusions of this study can be used to inform the design of wearable sensors which incorporate an algorithm to estimate RR from the ECG or PPG. Firstly, algorithms were typically more precise when using the ECG than the PPG, suggesting that preference should be given to using the ECG to estimate RR. Secondly, we recommend using a time-domain RR estimation technique, rather than a frequency-domain technique, since the best-performing algorithms used time-domain techniques. Thirdly, we recommend using a modulation fusion technique to fuse estimates corresponding to the three respiratory modulations, for the same reason.

Future work is required to investigate the generalisability of these conclusions. In particular, it is important to determine whether algorithm performance is significantly influenced by artifact, recording equipment and subjects’ physiology.

This study provides a publicly available toolbox of algorithms and data to enable future authors to perform reproducible and easily-compared assessments of novel algorithms. The toolbox of algorithms and data is available at: http://peterhcharlton.github.io/RRest. We welcome developments to the existing code or contributions of new algorithms for inclusion in future versions of the toolkit.

## Data access statement

The algorithms and data used in this research are openly available at http://peterhcharlton.github.io/RRest. The results presented here are available as supplementary material. Further information about the data and conditions of access can be found by emailing research.data@kcl.ac.uk.

## References

[pmeaaa1942bib001] Addison P S, Watson J N, Mestek M L, Mecca R S (2012). Developing an algorithm for pulse oximetry derived respiratory rate (RR(oxi)): a healthy volunteer study. J. Clin. Monit. Comput..

[pmeaaa1942bib002] Addison P S, Watson J N, Mestek M L, Ochs J P, Uribe A A, Bergese S D (2015). Pulse oximetry-derived respiratory rate in general care floor patients. J. Clin. Monit. Comput..

[pmeaaa1942bib003] Addison P, Watson J (2004). Secondary transform decoupling of shifted nonstationary signal modulation components: application to photoplethysmography. Int. J. Wavelets Multiresolution Inf. Process..

[pmeaaa1942bib004] Bailon R, Sornmo L, Laguna P, Clifford G D (2006). ECG-derived respiratory frequency estimation. Advanced Methods and Tools for ECG Data Analysis.

[pmeaaa1942bib005] Barnhart H X, Haber M J, Lin L I (2007). An overview on assessing agreement with continuous measurements. J. Biopharmaceutical Stat..

[pmeaaa1942bib006] Bonato P (2010). Wearable sensors and systems. IEEE Eng. Med. Biol. Mag..

[pmeaaa1942bib007] Braun S R, Walker H (1990). Respiratory rate and pattern. Clinical Methods: the History, Physical, and Laboratory Examinations.

[pmeaaa1942bib008] Carstensen B, Simpson J, Gurrin L C (2008). Statistical models for assessing agreement in method comparison studies with replicate measurements. Int. J. Biostat..

[pmeaaa1942bib009] Cretikos M A, Bellomo R, Hillman K, Chen J, Finfer S, Flabouris A (2008). Respiratory rate: the neglected vital sign. Med. J. Aust..

[pmeaaa1942bib010] Fleming S (2010). Measurement and fusion of non-invasive vital signs for routine triage of acute paediatric illness. PhD Thesis.

[pmeaaa1942bib011] Fleming S G, Tarassenko L (2006). A comparison of signal processing techniques for the extraction of breathing rate from the photoplethysmogram. Int. J. Biol. Life Sci..

[pmeaaa1942bib012] Fleming S, Tarassenko L, Thompson M, Mant D (2008). Non-invasive measurement of respiratory rate in children using the photoplethysmogram.

[pmeaaa1942bib013] Garbino A, Blue R S, Pattarini J M, Law J, Clark J B (2014). Physiological monitoring and analysis of a manned stratospheric balloon test program. Aviat. Space Environ. Med..

[pmeaaa1942bib014] Garde A, Karlen W, Ansermino J M, Dumont G A (2014). Estimating respiratory and heart rates from the correntropy spectral density of the photoplethysmogram. PLoS One.

[pmeaaa1942bib015] Gibbons R J (1997). ACC/AHA guidelines for exercise testing: a report of the American College of Cardiology/American Heart Association task force on practice guidelines (Committee on Exercise Testing). J. Am. Coll. Cardiol..

[pmeaaa1942bib016] Goldhill D R, White S A, Sumner A (1999). Physiological values and procedures in the 24 h before ICU admission from the ward. Anaesthesia.

[pmeaaa1942bib017] Hamilton P S, Tompkins W J (1986). Quantitative investigation of QRS detection rules using the MIT/BIH arrhythmia database. IEEE Trans. Biomed. Eng..

[pmeaaa1942bib018] Hao Y, Foster R (2008). Wireless body sensor networks for health-monitoring applications. Physiol. Meas..

[pmeaaa1942bib019] Hirsch J, Bishop B (1981). Respiratory sinus arrhythmia in humans: how breathing pattern modulates heart rate. Am. J. Physiol..

[pmeaaa1942bib020] Jeong J W, Jang Y W, Lee I, Shin S, Kim S, Magjarevic R (2009). Wearable respiratory rate monitoring using piezo-resistive fabric sensor. World Congress on Medical Physics and Biomedical Engineering.

[pmeaaa1942bib021] Johansson A (2003). Neural network for photoplethysmographic respiratory rate monitoring. Med. Biol. Eng. Comput..

[pmeaaa1942bib022] Johansson A, Strömberg T (2000). Influence of tidal volume and thoraco-abdominal separation on the respiratory induced variation of the photoplethysmogram. J. Clin. Monit. Comput..

[pmeaaa1942bib023] Karlen W, Ansermino J M, Dumont G (2012). Adaptive pulse segmentation and artifact detection in photoplethysmography for mobile applications.

[pmeaaa1942bib024] Karlen W, Raman S, Ansermino J M, Dumont G A (2013). Multiparameter respiratory rate estimation from the photoplethysmogram. IEEE Trans. Biomed. Eng..

[pmeaaa1942bib025] Lapi S, Lavorini F, Borgioli G, Calzolai M, Masotti L, Pistolesi M, Fontana G (2014). Respiratory rate assessments using a dual-accelerometer device. Respiratory Physiol. Neurobiol..

[pmeaaa1942bib026] Lázaro J, Gil E, Bailón R, Mincholé A, Laguna P (2013). Deriving respiration from photoplethysmographic pulse width. Med. Biol. Eng. Comput..

[pmeaaa1942bib027] Lázaro Plaza J (2015). Non-invasive techniques for respiratory information extraction based on pulse photoplethysmogram and electrocardiogram. PhD Thesis.

[pmeaaa1942bib028] Lee J, Chon K H (2010). An autoregressive model-based particle filtering algorithms for extraction of respiratory rates as high as 90 breaths per minute from pulse oximeter. IEEE Trans. Biomed. Eng..

[pmeaaa1942bib029] Lindberg L G, Ugnell H and Öberg P Å. 1992 Monitoring of respiratory and heart rates using a fibre-optic sensor. Med. Biol. Eng. Comput..

[pmeaaa1942bib031] Mateo J, Laguna P (2003). Analysis of heart rate variability in the presence of ectopic beats using the heart timing signal. IEEE Trans. Biomed. Eng..

[pmeaaa1942bib032] Meredith D J, Clifton D, Charlton P, Brooks J, Pugh C W, Tarassenko L (2012). Photoplethysmographic derivation of respiratory rate: a review of relevant physiology. J. Med. Eng. Technol..

[pmeaaa1942bib033] Moll J M, Wright V (1972). An objective clinical study of chest expansion. Ann. Rheumatic Diseases.

[pmeaaa1942bib034] Moody G B, Mark R G, Zoccola A, Mantero S (1985). Derivation of respiratory signals from multi-lead ECGs. Conf. Proc. CinC..

[pmeaaa1942bib035] Nemati S, Malhotra A, Clifford G D (2010). Data fusion for improved respiration rate estimation. EURASIP J. Adv. Signal Process..

[pmeaaa1942bib036] O’Brien I, O’Hare P, Corrall R (1986). Heart rate variability in healthy subjects: effect of age and the derivation of normal ranges for tests of autonomic function. Br. Heart J..

[pmeaaa1942bib037] Orphanidou C, Bonnici T, Charlton P, Clifton D, Vallance D, Tarassenko L (2015). Signal-quality indices for the electrocardiogram and photoplethysmogram: derivation and applications to wireless monitoring. IEEE J. Biomed. Health Inf..

[pmeaaa1942bib038] Orphanidou C, Clifton D, Khan S, Smith M, Feldmar J, Tarassenko L (2009b). Telemetry-based vital sign monitoring for ambulatory hospital patients.

[pmeaaa1942bib039] Orphanidou C, Brain O, Feldmar J, Khan S, Price J, Tarassenko L (2009a). Spectral fusion for estimating respiratory rate from the ECG. Conf. Proc. ITAB.

[pmeaaa1942bib040] Orphanidou C, Fleming S, Shah S, Tarassenko L (2013). Data fusion for estimating respiratory rate from a single-lead ECG. Biomed. Signal Process. Control.

[pmeaaa1942bib041] Pan J, Tompkins W J (1985). A real-time QRS detection algorithm. IEEE Trans. Biomed. Eng..

[pmeaaa1942bib042] Pimentel M A F, Charlton P H, Clifton D A, Mukhopadhyay S C (2015). Probabilistic estimation of respiratory rate from wearable sensors. Wearable Electronics Sensors.

[pmeaaa1942bib043] Rajkumar K, Ramya K (2013). Respiration rate diagnosis using single lead ECG in real time. Glob. J. Med. Res..

[pmeaaa1942bib044] Ridley S (2005). The recognition and early management of critical illness. Ann. R. Coll. Surg. Engl..

[pmeaaa1942bib045] Royal College of Physicians (2012). National Early Warning Score (NEWS): Standardising the assessment of acute-illness severity in the NHS. Technical Report.

[pmeaaa1942bib046] Ruangsuwana R, Velikic G, Bocko M (2010). Methods to extract respiration information from ECG signals. Conf. Proc. ICASSP.

[pmeaaa1942bib047] Schäfer A, Kratky K W (2008). Estimation of breathing rate from respiratory sinus arrhythmia: comparison of various methods. Ann. Biomed. Eng..

[pmeaaa1942bib048] Schein R M, Hazday N, Pena M, Ruben B H, Sprung C L (1990). Clinical antecedents to in-hospital cardiopulmonary arrest. Chest.

[pmeaaa1942bib049] Shah S A (2012). Vital sign monitoring and data fusion for paediatric triage. PhD Thesis.

[pmeaaa1942bib050] Shah S A, Fleming S, Thompson M, Tarassenko L (2015). Respiratory rate estimation during triage of children in hospitals. J. Med. Eng. Technol..

[pmeaaa1942bib051] Sobron A, Romero I, Lopetegi T (2010). Evaluation of methods for estimation of respiratory frequency from the ECG. Conf. Proc. CinC.

[pmeaaa1942bib052] Thayer J, Sollers J, Ruiz-Padial E, Vila J (2002). Estimating respiratory frequency from autoregressive spectral analysis of heart period. IEEE Eng. Med. Biol. Mag..

[pmeaaa1942bib053] Widjaja D, Varon C, Dorado A C, Suykens J A K, Van Huffel S (2012). Application of kernel principal component analysis for single-lead-ECG-derived respiration. IEEE Trans. Biomed. Eng..

[pmeaaa1942bib054] Yilmaz T, Foster R, Hao Y (2010). Detecting vital signs with wearable wireless sensors. Sensors.

